# Measuring the Healthiness of Ready-to-Eat Child-Targeted Cereals: Evaluation of the FoodSwitch Platform in Sweden

**DOI:** 10.2196/17780

**Published:** 2021-07-22

**Authors:** Antoine Mottas, Veli-Matti Lappi, Johan Sundström, Bruce Neal, Cliona Ni Mhurchu, Marie Löf, Karin Rådholm

**Affiliations:** 1 Department of Biosciences and Nutrition Karolinska Institutet Stockholm Sweden; 2 Department of Medical Sciences Uppsala University Uppsala Sweden; 3 The George Institute for Global Health University of New South Wales Sydney Australia; 4 Department of Epidemiology and Biostatistics Imperial College London London United Kingdom; 5 National Institute for Health Innovation School of Population Health University of Auckland Auckland New Zealand; 6 Department of Health, Medicine and Caring Sciences Linköping University Linköping Sweden

**Keywords:** breakfast cereals, child-targeted cereals, front-of-pack labels, Keyhole symbol, Health Star Rating, FoodSwitch, diet, food intake

## Abstract

**Background:**

Childhood obesity is a major public health issue. The increase in the consumption of foods with poor nutritional value, such as processed foods, contributes to this. Breakfast cereals are often advertised as a healthy way to start the day, but the healthiness of these products varies greatly.

**Objective:**

Our main objective was to gather information about the nutritional characteristics of ready-to-eat breakfast cereals in Sweden and to investigate the healthiness of products targeted at children compared to other cereals by use of the FoodSwitch platform. A secondary objective was to evaluate the alignment between the Keyhole symbol and the Health Star Rating.

**Methods:**

The FoodSwitch app is a mobile health (mHealth) tool used to present nutrition data and healthier alternative products to consumers. Ready-to-eat breakfast cereals from the largest Swedish grocery retailers were collected using the FoodSwitch platform. Products were defined as targeting children if they presented features addressing children on the package.

**Results:**

Overall, information on 261 ready-to-eat cereals was examined. Of this total, 8% (n=21) were targeted at children. Child-targeted cereals were higher in sugar (22.3 g/100 g vs 12.8 g/100 g, *P*<.001) and lower in fiber (6.2 g/100 g vs 9.8 g/100 g, *P*<.001) and protein (8.1 g/100 g vs 10.5 g/100 g, *P*<.001). Total fat (3 g/100 g vs 10.5 g/100 g, *P*<.001) and saturated fat (0.8 g/100 g vs 2.6 g/100 g, *P*<.001) were also lower. No difference was found in salt content (*P*=.61). Fewer child-targeted breakfast cereals displayed an on-pack Keyhole label (n=1, 5% vs n=53, 22%; *P*=.06), and the mean Health Star Rating value was 3.5 for child-targeted cereals compared to others (mean 3.8, *P*=.07). A correlation was found between the Keyhole symbol and the Health Star Rating.

**Conclusions:**

Ready-to-eat breakfast cereals targeted at children were less healthy in terms of sugar and fiber content compared to products not targeted at children. There is a need to improve the nutritional quality of child-targeted cereals.

## Introduction

Childhood obesity is a major public health issue. Over 40 million children under 5 years of age are overweight worldwide with a majority living in low- or middle-income countries [1]. The increasing rates of obesity in children are linked to noncommunicable diseases such as type 2 diabetes and cardiovascular diseases in young adults [2]. In addition, individuals with obesity at an early age are more prone to obesity later in life [3]. Unhealthy diet is the leading risk factor for obesity in children and adults. Processed foods, which are energy dense and of poor nutritional value, are a major cause of obesity [4]. In the last few decades, the consumption of processed and ultraprocessed food has increased and correlates with the elevated prevalence of obesity [5].

Ready-to-eat breakfast cereals include a large range of products from unprocessed to ultraprocessed. This large diversity in products and the omnipresence of nutritional claims on the packages of breakfast cereals make it hard for the consumer to identify actual healthy options. In addition, there are data suggesting that highly advertised products targeted at children may be less healthy [6]. To help consumers, front-of-pack nutrition labels have been created. There are many types of labeling, often specific to a country or area, but all aim to promote healthier food choices and, in the long term, to urge manufacturers to reformulate their products [7]. The Swedish National Food Administration introduced the Keyhole symbol that endorses products with healthier fat composition, less sugars and salt, and more dietary fiber, whole grains, fruits, and vegetables than other similar foods [8]. The Health Star Rating (HSR), developed by the Australian government, grades foods from 0.5 to 5 stars [9]. Grades are attributed based on energy content, saturated fat, sugar, salt, protein, fiber, as well as fruit, vegetable, nut, and legume content, in the product. In other countries different front-of-pack labels are used. For instance, nutrient-specific labels that assess the percentage of each nutrients (eg, Traffic Lights labeling and nutrient-specific warnings that indicate an excessive amount of critical nutrients). The weakness of most systems is that they remain voluntary and rely on industry interest. In addition, the coexistence of numerous front-of-pack labels in the marketplace, as well as the great disparity between their criteria, can be confusing for consumers [10].

The use of mobile apps could be an innovative alternative way for consumers to access more information on the nutrients and healthiness of the foods they buy. Today, 88% of people in Sweden over the age of 12 years own a smartphone, which offers great potential for mobile health (mHealth) apps in all age groups [11]. The FoodSwitch app, developed by the George Institute for Global Health in Australia, was designed to offer a tool to promote healthier food choices and is currently being used in Australia, New Zealand, China, the United Kingdom, India, the United States, Kuwait, South Africa, Fiji, and Hong Kong. By scanning bar codes of packaged foods, the consumer obtains at-a-glance information on the product as well as suggestions of similar healthier products [12]. The FoodSwitch solution includes the FoodSwitch app, a database with packaged products organized in a categorized system that is applicable to all countries and enables comparisons, and a data collection application (DataCollector). The FoodSwitch database has been previously used in several studies to measure the healthiness of packaged food, to evaluate variations in specific nutrients, and to compare front-of-pack labeling systems [13-15].

In this study, we aim to use the FoodSwitch platform in the Swedish market to compare the healthiness and nutritional values of ready-to-eat breakfast cereals targeted at children to non–child-targeted, ready-to-eat cereals. Furthermore, we aim to assess the alignment of the Nordic Keyhole symbol and the HSR system for these products.

## Methods

### Data Collection

Ready-to-eat cereals from 4 supermarkets (ICA, Coop, Hemköp, and Lidl), representing 90% of the market share in Sweden, were analyzed [16]. The largest supermarkets in the Stockholm area were targeted to ensure the presence of the entire range of products. Prior to data collection, which took place in February and March 2019, consent forms were sent to the store managers. One store denied us permission without giving more details on why they chose to do so, and thus this retailer was not included. On site, the cereal department and all shelves containing breakfast cereals, including gluten-free sections, were identified. Oatmeal for porridge was regarded as a grain and not used as a breakfast cereal per se. Muesli, however, was included. There is a thin line between cereal bars and candy, and cereal bars are categorized differently from breakfast cereal on the FoodSwitch platform. Thus, they were not deemed breakfast cereal and were excluded.

The DataCollector app of the FoodSwitch platform (Android, version 2.7) from the George Institute for Global Health was used to collect information from packages. First, the bar codes were scanned using a smartphone camera. Then, numerous pictures of the front of the pack, the Nutrition Information Panel (NIP), and the ingredients were taken to collect all relevant information.

All products were manually entered in the system by 1 researcher (AM). The NIP, the gluten status and the Keyhole symbol, if present, were recorded. All child-targeted information on the package was identified. The criteria were as follows: the presence of cartoons, games, toys, children’s movie references, or text addressed to children on the package. A second researcher (V-ML) reviewed all the information entered and confirmed the presence of marketing targeted at children.

Finally, products were categorized and an HSR was generated. HSR scores of 0.5 to 5 stars in 10 half-star increments were assigned to all scanned products, where a higher number of stars represents healthier products. In the HSR system, each packaged food item is categorized into 1 of 6 categories depending on food type. All breakfast cereals were assigned to category 2. The HSR score was calculated via baseline and modifying points using the following formula: HSR score = baseline points – modifying points. Baseline points depended on energy content, saturated fat, sugar, and salt, and modifying points were based on protein, fiber, fruit, and vegetable content. The final assignment of the HSR score depended on which category the product was assigned to [17].

### Statistical Analysis

Categorical variables were summarized as the number of products and corresponding percentages, and continuous variables were summarized as mean (SD) and median (IQR). A Kolmogorov-Smirnov test was used to determine whether the data were normally distributed or not. A Mann-Whitney *U* test and an independent-samples *t* test were used to compare the nutritional values and HSR of child-targeted cereals to the nontargeted ones for nonnormal and normal distribution, respectively. In addition, the HSR scores were divided into two groups, healthier (HSR≥3.5) and less healthy (HSR<3.5) [18]. After dichotomization of HSR scores, the Fisher exact test was performed to assess correlation to the presence of the Keyhole symbol. The significance level was set to *P*<.05. The statistical analysis was performed using SPSS Statistics 25 (IBM Corp).

### Ethical Considerations

This was a study of packaged food supplies in supermarkets in Sweden and did not involve study participants or animal testing. Therefore, no ethical permission was sought.

## Results

By use of the data collection application of the FoodSwitch platform, we collected information on a total of 261 ready-to-eat breakfast cereals, of which 21 (8%) were targeted at children and 240 (92%) were not. Table 1 summarizes the nutritional content from the NIP of all products as well as their HSR score and the number of products displaying the Keyhole symbol. Child-targeted cereals contained more sugars, with a mean carbohydrate content of 78.3 g compared to 62.0 g per 100 g for the non–child-targeted group (*P*<.001). This applied to sugar as well, where the mean in child-targeted products was nearly twice as high than in the non–child-targeted ones (22.3 g vs 12.8 g per 100 g, *P*<.001). Total fat, saturated fat, fiber, and protein were all lower in the child-targeted cereals compared to the cereals not targeted at children (*P*<.001 for all). There was no difference in salt content (*P*=.61) between the groups. The main categories of cereals not targeted at children were muesli and granola, while cocoa-based and sweetened cereals accounted for the majority of child-targeted cereals (Table S1 in Multimedia Appendix 1).

**Figure 1:**
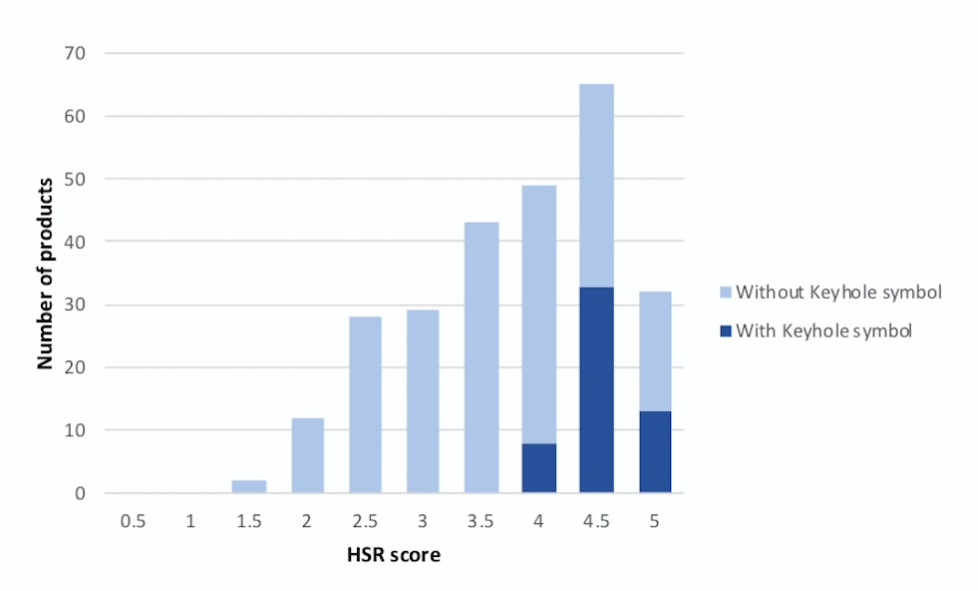
The calculated Health Star Rating (HSR) score of breakfast cereals in supermarkets in Sweden (light blue) and the HSR scores of packaged products displaying an on-pack Keyhole symbol (dark blue).

Figure 1 shows that on-pack Keyhole labeling was aligned with products considered as “healthy” (≥3.5) according to the HSR (*P*<.001). No products with an HSR score <4 were labeled with the Keyhole symbol. However, 60% (n=92) of products with 4 stars or more were not Keyhole labeled. According to the HSR, there was no significant difference between cereals targeted at children and those that were not (mean 3.5 for the child-targeted group vs 3.8 for the non–child-targeted group, *P*=.07).

**Table 1:** Nutritional overview of ready-to-eat breakfast cereals targeted at children (n=21) compared to those not targeted at children (n=240) and their estimated healthiness. The mean fiber amount in child-targeted cereals was based on 20 products since 1 product did not display its fiber content.

Nutrition^a^		Child-targeted cereals	Non–child-targeted cereals	*P* value^b^
**Energy (kJ/100 g)**				.18
	Mean (SD)	1628 (68)	1699 (202)	
	Median (IQR)	1618 (49)	1647 (225)	
**Total fat (g/100 g)**				<.001
	Mean (SD)	3.0 (2.8)	10.5 (9.6)	
	Median (IQR)	2.3 (1.5)	7.6 (12.8)	
**Saturated fat (g/100 g)**				<.001
	Mean (SD)	0.8 (0.8)	2.6 (3.0)	
	Median (IQR)	0.6 (0.6)	1.8 (3.0)	
**Carbohydrates (g/100 g)**				<.001
	Mean (SD)	78.3 (5.3)	62.0 (13.5)	
	Median (IQR)	78.0 (6.1)	62.0 (13.4)	
**Sugars (g/100 g)**				<.001
	Mean (SD)	22.3 (7.5)	12.8 (7.8)	
	Median (IQR)	23.5 (13.0)	11.0 (12.1)	
**Protein (g/100 g)**				.002
	Mean (SD)	8.1 (1.7)	10.5 (2.6)	
	Median (IQR)	8.2 (2.2	10.0 (3.2))	
**Fiber (g/100 g)**				.01
	Mean (SD)	6.2 (2.5)	9.8 (5.0)	
	Median (IQR)	6.8 (3.6)	9.0 (4.8)	
**Salt (g/100 g)**				.61
	Mean (SD)	0.5 (0.3)	0.5 (0.4)	
	Median (IQR)	0.6 (0.6)	0.4 (0.6)	
**HSR^c^ score**				.07
	Mean (SD)	3.5 (0.5)	3.8 (0.9)	
	Median (IQR)	3.8 (1.0)	4.0 (1.5)	
Keyhole symbol, n (%)		1 (5)	53 (22)	.06

^a^Data are n (%) for the categorical variable and mean (SD) and median (IQR) for continuous variables.

^b^Comparison of child-targeted versus non–child-targeted breakfast cereals was analyzed with a Mann-Whitney *U* test.

^c^HSR: Health Score Rating.

## Discussion

### Principal Findings

This study compared the healthiness of ready-to-eat breakfast cereals targeted at children to cereal products not specifically targeted at children across supermarkets in Sweden using the FoodSwitch platform. Despite lower levels of saturated fat, the child-targeted cereals were overall less healthy, according to the nutritional content. Child-targeted products had a greater amount of sugar, with a mean nearly 2 times higher than the cereals not branded toward children. Furthermore, they were lower in fiber and protein. Our results are in line with 3 similar studies conducted in Canada, Australia, New Zealand, Guatemala, and the United States [19-23]. However, this is the first study focusing on the healthiness of packaged cereal products in Europe. Interestingly, the number of products targeted at children is relatively small. Compared to the markets analyzed in other countries, we obtained the smallest ratio between child-targeted cereals and non–child-targeted ones [19-23]. A few cereals rated high in healthiness according to the HSR system although these had a high sugar content. This aligns with previous findings showing that the HSR rating algorithm does not sufficiently penalize products with a high added sugar content [24].

### Societal Relevance

In a context where childhood obesity is at the heart of public health concerns, our findings are highly relevant. The direct link between added sugar intake and obesity is well established [25,26]. Recently, the American Heart Association reported associations between added sugars and cardiovascular disease risk factors among children at levels well below the actual average intake [27]. Therefore, sugar levels in foods intended for children should be as low as possible or at least be similar to products not branded toward children. Dietary fibers are known to improve satiety, and fiber consumption is associated with lower body weight [28]. Even though it has been shown that consumers of breakfast cereals are more likely to reach the recommended daily amount of fiber, there should be no difference in fiber between food items branded toward children and cereals not targeted at children [29]. The presence of more total fat and saturated fat in non–child-targeted products was probably due to cereal type. When the products were categorized, we noticed that granola and muesli accounted for a majority. These types of cereals often contain nuts and seeds, which are high in fat. Salt content was similarly low in all products. In fact, many sodium-reduction initiatives have been introduced as part of the European Union salt reduction framework [30]. One study showed a significant drop in salt levels in breakfast cereals in the United Kingdom [31]. We can assume that the same happened in Sweden since the brands sold in both countries are very similar; however, this would be possible to investigate in the future by using this study as the reference values for 2019.

Marketing toward children often depicts cartoon characters to influence their food preferences [32]. In addition, children exposed to child-targeted cereals via TV commercials tend to have higher intake of these advertised cereals [33]. Problematically, products advertised to children are often of poor nutritional quality, as demonstrated in our study [34]. Unfortunately, packaging also affects parents, and parents being role models influence the eating habits of their children [35]. In fact, children depend on their parents’ food choices. Finally, it has been shown that NIPs and front-of-pack labels can sometimes be difficult to interpret [36]. Therefore, there is a need to provide easy-to-understand solutions to better guide consumers and implement healthy dietary habits from the earliest age.

### Potential for the FoodSwitch Platform in Sweden

A substantial part of the foods sold and eaten in high-income countries is preprepared packaged foods, and processed foods are largely responsible for an excessive intake of saturated fat, energy, added sugars, and sodium in the diet [37]. Thus, the food industry plays an increasingly important role in public health. The aim of the FoodSwitch platform is to change consumers’ behaviors and legislators’ actions. Front-of-pack labels are well established in influencing consumers toward healthier food choices. The Keyhole symbol used in Sweden was implemented 30 years ago. This front-of-pack logo is well known by Swedes as well as Scandinavians [38]. Although it is hard to assess its effect, it seems to positively affect the consumers’ behavior [39]. Unfortunately, not all manufacturers use the logo even though it is free of charge. The criteria required to make a product eligible for the Keyhole symbol are numerous and vary according to product type. Thus, producers might not be aware when a product meets the standard. Moreover, displaying the symbol induces extra costs for new packaging. Manufacturers often prioritize the sales of their foods. Therefore, another reason for the lack of compliance to the Keyhole symbol might be that producers are not convinced of any added value by the logo on their products. Throughout our investigation, we realized that many products considered as “healthy” by the HSR system (>3.5) did not display the Keyhole symbol. It is very likely that some of them were suitable to be branded with the Keyhole symbol.

**Figure 2:**
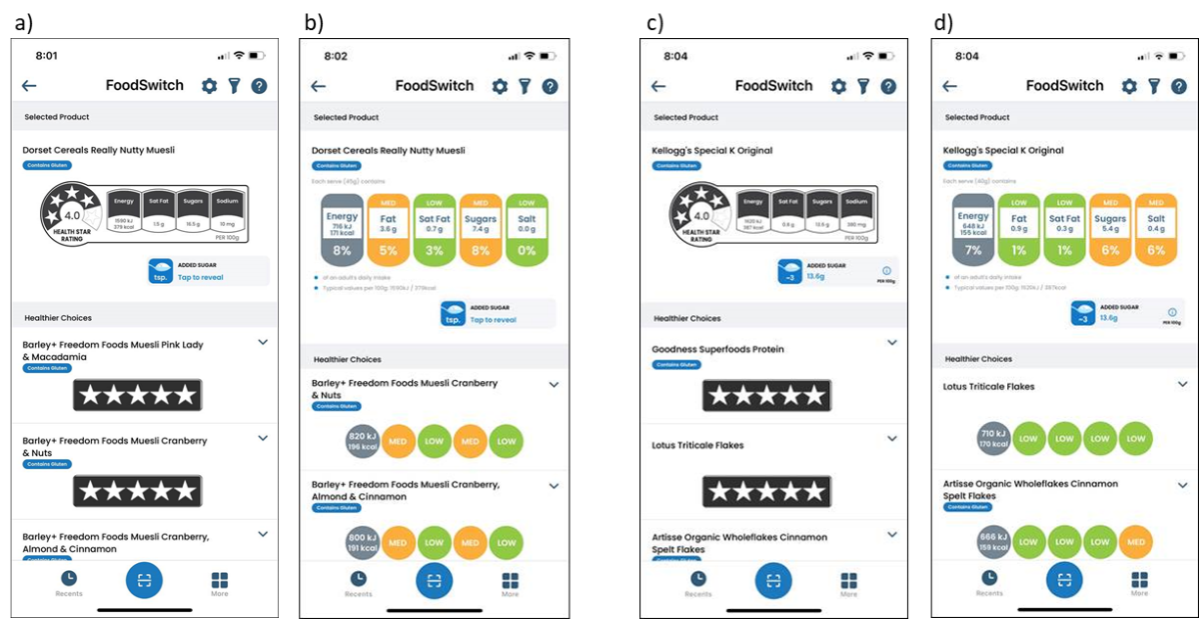
Screenshots of the Australian version of the FoodSwitch app displaying the estimated healthiness of products using Health Star Ratings (panels A and C) and Traffic Lights ratings (panels B and D) for the scanned products Dorset Cereals Really Nutty Muesli (panels A and B) and Kellogg’s Special K Original (panels C and D), and suggestions for healthier options.

Because of this, introducing the FoodSwitch app in Sweden could be of interest to Swedish consumers. First, it would allow a virtual front-of-pack labeling of all products (Figure 2). In fact, with information from the database, the app could generate the HSR score and the Traffic Lights label for any packaged product and suggest a healthier alternative. However, these two front-of-pack labeling systems used in the FoodSwitch app to direct consumers toward a healthier food choice are not used in Sweden. Fortunately, both the HSR and Traffic Lights label are self-explanatory and should not impede compliance of Swedish users to the mobile app’s recommendations. In addition, it would be possible to add a filter on the app that would generate the Keyhole symbol. Second, the implementation of FoodSwitch in Sweden could pressure manufacturers to reformulate their products. If consumers choose healthier packaged foods, producers would have to adapt to evolving client demand. Finally, the FoodSwitch database would be a very useful tool for conducting research on many aspects of the packaged food market. Through crowdsourcing, the information from packaged foods are always updated, which allows a tracking of the nutritional composition of the food supply over time.

### Strengths, Limitations, and Future Directions

We consider the conclusions drawn from our primary investigation to be reliable for the Swedish market because we visited the largest grocery retailers, representing 90% of market shares [16]. Another strength is the reliability of our data. During data collection, the data were double-checked by the collector prior to data entry. Once entered, the data were checked by a second researcher.

Limitations include possible transcribing errors and, in some cases, minor discrepancies that can occur between the NIP information shown on the package and the real value [40]. In terms of the method of assessing the healthiness of products, in Europe, displaying fiber content on the package is not mandatory [41]; however, it is often included on the NIP and only one product in our sample did not display fiber content. Finally, another limitation is that our results are limited to the Swedish market.

A further research objective could be to expand this assessment of nutrition to all child-targeted products. Additionally, data collection of breakfast cereals can be redone to provide a longitudinal perspective.

### Conclusion

In conclusion, we showed that the nutritional quality of ready-to-eat cereals targeted at children was overall not significantly unhealthier than ready-to-eat breakfast cereals not targeted at children. However, cereals targeted at children were high in sugar and low in fiber. Thus, we conclude that there is a need to improve the dietary quality of child-targeted breakfast cereals in Sweden.

## Appendix

Multimedia Appendix 1 Ready-to-eat breakfast cereals in Swedish supermarkets in 2019, categorized according to the FoodSwitch platform categories.

## Abbreviations

HSR: Health Star Rating
mHealth: mobile health
NIP: Nutrition Information Panel
