# Supplementary material for: Measuring the Healthiness of Ready-to-Eat Child-Targeted Cereals: Evaluation of the FoodSwitch Platform in Sweden
Source: JMIR Mhealth Uhealth. 2021 Jul 22;9(7):e17780. doi: 10.2196/17780 (PMC8367182; doi:10.2196/17780)
Supplement: Multimedia Appendix 1 [file mhealth_v9i7e17780_app1.docx]

### Supplementary Table 1

Ready-to-eat breakfast cereals in Swedish supermarkets in 2019, categorized according to the FoodSwitch platform categories.

| Categories | | Frequency | Percent |
| --- | --- | --- | --- |
|  | Breakfast cereal variety packs | 1 | 4.8 |
|  | Cocoa pop style cereal | 1 | 4.8 |
|  | Cocoa-based cereal | 9 | 42.9 |
|  | Corn flakes | 2 | 9.5 |
|  | Other puffed cereals not otherwise specified | 2 | 9.5 |
|  | Other sweet style cereal not otherwise specified | 5 | 23.8 |
|  | Rice bubbles | 1 | 4.8 |
|  | Total | 21 | 100.0 |

Ready-to-eat breakfast cereals targeted at children.

| Categories | | Frequency | Percent |
| --- | --- | --- | --- |
|  | Brans with additions | 4 | 1.7 |
|  | Breakfast biscuits (e.g. Weet-Bix) | 1 | .4 |
|  | Breakfast bites | 7 | 2.9 |
|  | Cocoa-based cereal | 1 | .4 |
|  | Corn flakes | 18 | 7.5 |
|  | Flakes with additions | 14 | 5.8 |
|  | Flavoured oats | 4 | 1.7 |
|  | Granola/cluster cereals | 47 | 19.6 |
|  | Muesli with fruit | 66 | 27.5 |
|  | Other breakfast cereal products not otherwise specified | 36 | 15.0 |
|  | Other plain flakes not otherwise specified | 18 | 7.5 |
|  | Other puffed cereals not otherwise specified | 2 | .8 |
|  | Plain brans | 10 | 4.2 |
|  | Plain muesli | 10 | 4.2 |
|  | Rice bubbles | 2 | .8 |
|  | Total | 240 | 100.0 |

Ready-to-eat breakfast cereal not targeted at children.
